# Effects of Sodium-Glucose Co-Transporter-2 Inhibitors on Pancreatic β-Cell Mass and Function

**DOI:** 10.3390/ijms23095104

**Published:** 2022-05-04

**Authors:** Akinobu Nakamura

**Affiliations:** Department of Rheumatology, Endocrinology and Nephrology, Faculty of Medicine, Graduate School of Medicine, Hokkaido University, Sapporo 060-8638, Japan; akinbo@tim.hi-ho.ne.jp; Tel.: +81-11-706-5915

**Keywords:** beta-cells, glucokinase, glucose metabolism, insulin secretion

## Abstract

Sodium-glucose co-transporter-2 inhibitors (SGLT2is) not only have antihyperglycemic effects and are associated with a low risk of hypoglycemia but also have protective effects in organs, including the heart and kidneys. The pathophysiology of diabetes involves chronic hyperglycemia, which causes excessive demands on pancreatic β-cells, ultimately leading to decreases in β-cell mass and function. Because SGLT2is ameliorate hyperglycemia without acting directly on β-cells, they are thought to prevent β-cell failure by reducing glucose overload in this cell type. Several studies have shown that treatment with an SGLT2i increases β-cell proliferation and/or reduces β-cell apoptosis, resulting in the preservation of β-cell mass in animal models of diabetes. In addition, many clinical trials have shown that that SGLT2is improve β-cell function in individuals with type 2 diabetes. In this review, the preclinical and clinical data regarding the effects of SGLT2is on pancreatic β-cell mass and function are summarized and the protective effect of SGLT2is in β-cells is discussed.

## 1. Introduction

In 2021, 540 million people around the world between the ages of 20 and 79 were estimated to have diabetes, and this number is expected to increase further [1]. Most of these individuals have type 2 diabetes (T2D). The pathophysiology of T2D is characterized by insulin resistance and a reduction in insulin secretion, with the latter playing important roles in both the onset and progression of the disease [2,3,4,5]. The progressive decrease in insulin secretion is the result of pancreatic β-cell failure, involving a loss of β-cell mass and an impairment in β-cell function [6,7,8]. Therefore, the prevention of β-cell failure by the preservation of β-cell mass and function would prevent the progression of T2D.

Sodium-glucose co-transporter-2 inhibitors (SGLT2is) are oral antihyperglycemic agents that reduce the blood glucose concentration by inhibiting glucose reabsorption in the proximal tubules of the kidney and promoting its excretion, causing glucosuria [9]. In addition, their use is associated with a low risk of hypoglycemia, and they have protective effects in organs, including the heart and kidneys [9]. Since the publication of the findings of the EMPA-REG OUTCOME trial in 2015, randomized controlled trials and meta-analyses have shown that treatment with an SGLT2i has secondary preventive effects on major cardiovascular events in individuals with T2D, and has preventive effects on hospitalization for heart failure (HF) and the progression to end-stage renal disease in individuals with T2D, regardless of the presence or absence of atherosclerotic cardiovascular disease (ASCVD) or a history of HF [10,11,12,13,14]. Therefore, the Standards of Medical Care in Diabetes by the American Diabetes Association state that an SGLT2i is an appropriate initial therapy for individuals with T2D who are at high risk for ASCVD, HF, and/or chronic kidney disease [15].

This review summarizes the published preclinical and clinical data regarding the effects of SGLT2is on pancreatic β-cell mass and function and discusses the use of SGLT2is for the protection of β-cells.

## 2. SGLT2is Protect Pancreatic β-Cells

Chronic hyperglycemia is associated with macrovascular and microvascular complications in diabetes. Multifactorial interventions targeting blood pressure and the circulating lipid profile, and glycemic control, are important means of preventing and slowing the progression of macrovascular complications, including ASCVD [16,17,18]. On the other hand, intensive glycemic control itself delays the onset and slows the progression of microvascular complications, such as diabetic retinopathy and diabetic kidney disease [19,20,21], which implies that chronic hyperglycemia significantly contributes to the injury of the organs affected by microvascular complications.

**Figure 1:**
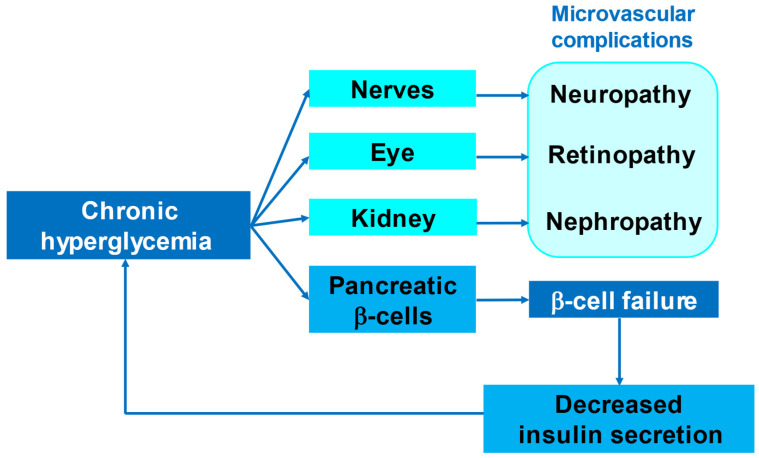
Target organs of the deleterious effects of chronic hyperglycemia.

Chronic hyperglycemia also affects pancreatic β-cells (Figure 1). Under normal circumstances, when the blood glucose concentration increases, insulin secretion by β-cells is increased, which reduces the blood glucose concentration. However, if the hyperglycemia is chronic, β-cells become overloaded, resulting in decreases in β-cell mass and function [22]. Because insulin is the only hormone that reduces the blood glucose concentration, if the secretion of insulin is insufficient, chronic hyperglycemia results. Persistently high concentrations of glucose have deleterious effects on β-cell mass and function, ultimately resulting in β-cell failure and further hyperglycemia (Figure 1). This vicious cycle is thought to mediate the progressive decline in β-cell mass and function that is an important feature of the pathophysiology of T2D.

A number of mechanisms have been proposed to explain how chronic hyperglycemia induces β-cell failure, but it is thought that metabolic stress, including oxidative stress and endoplasmic reticulum stress, plays an important role [22,23,24,25]. β-cells not only in *db/db* mice, which represent a slightly extreme model of obesity and T2D, but also in individuals with diabetes show a progressive reduction in mass that is associated with oxidative stress when exposed to long-term hyperglycemia [23,24,26]. A number of biochemical pathways through which hyperglycemia may cause excessive production of reactive oxygen species (ROS), which cause oxidative stress, have been identified. When metabolites of glucose are subjected to oxidative phosphorylation, ROS are produced at the same time as ATP is generated by the mitochondrial respiratory chain. Therefore, in glucose excess, the production of ROS increases. Additionally, some of these metabolites enter alternative pathways, such as hexosamine metabolism and sorbitol metabolism, which also result in ROS production [24,25]. ROS accumulation leads to β-cell failure through mitochondrial injury and lower expression of β-cell-associated transcription factors [27,28]. The role of metabolic stress induced by excessive glucose metabolism has been demonstrated using a transgenic mouse model of β-cell-specific genetic activation of glucokinase, the rate-limiting enzyme of glycolysis, the first step in glucose metabolism in β-cells. These mice develop β-cell failure as a result of greater metabolic stress and DNA damage [29]. In addition, mice with a heterozygous mutation that results in the activation of glucokinase in β-cells exhibit long-term glucose intolerance and lower β-cell mass [30]. These findings are consistent with the importance of excess glucose metabolism for the development of β-cell failure.

**Figure 2:**
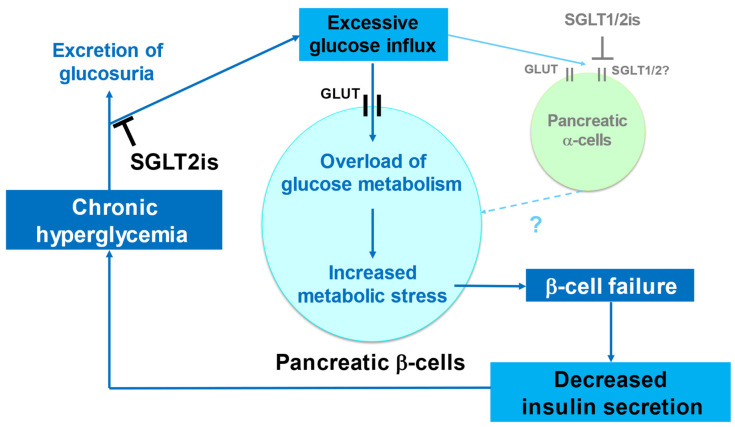
Basis for the use of sodium-glucose co-transporter-2 inhibitors (SGLT2is) to prevent the progression of pancreatic β-cell failure.

A reduction in the glucose overload of β-cells might break the vicious cycle described above (Figure 2). One strategy would be to reduce glucose metabolism in β-cells. To assess the potential for this approach, we generated *db/db* mice with glucokinase haploinsufficiency [31]. In these mice, the β-cell deficiency of glucokinase prevented the progressive decline in β-cell mass and function by reducing metabolic stress. Specifically, they showed higher expression of β-cell-associated transcription factors, such as *Mafa* and *Nkx6.1*, which results in improvements in insulin secretion and glucose tolerance [28,31]. Another strategy is to reduce excessive glucose influx into β-cells. SGLT2is have long-term antihyperglycemic effects [9]. The mechanism of action of SGLT2i is to promote the excretion of glucosuria via suppression of renal glucose reabsorption by inhibiting SGLT2. SGLT2 is one of the glucose transporters expressed in the proximal tubules of the kidney. In pancreatic β-cells, the glucose transporter (GLUT) family, such as GLUT1 and GLUT2, is responsible for glucose uptake into cells, and SGLT2 is not expressed [32,33,34,35,36,37]. Additionally, SGLT2is do not directly affect insulin secretion in the islets of rodents and humans [34,36]. Therefore, SGLT2is might reduce the excessive influx of glucose indirectly, and thereby prevent β-cell failure by reducing local glucose overload (Figure 2).

## 3. Findings Obtained from Animal Models Regarding the Effects of SGLT2is on Pancreatic β-Cell Mass and Function

As described above, although *db/db* mice represent a slightly extreme model of obesity and T2D, they demonstrate decreases in β-cell mass and function with advancing age [38], and therefore represent a good model in which to determine the protective effects on β-cell mass and the function of pharmacological interventions. Jurczak et al. established SGLT2 knockout mice on a *db/db* background [39], and found that their glucose tolerance, glucose-stimulated insulin secretion during a hyperglycemic clamp, and β-cell mass were higher than those of control *db/db* mice. These findings are consistent with a beneficial effect of SGLT2is on β-cell function. Indeed, several SGLT2is have been shown to have protective effects on β-cell mass and function in *db/db* mice [40,41,42,43] and in Zucker diabetic fatty rats, which also show similar progressive decreases in β-cell mass and function with age [44,45,46].

β-cell mass is determined by the levels of β-cell proliferation, apoptosis, and dedifferentiation that occur [47]. In *db/db* mice, SGLT2 deficiency or treatment with luseogliflozin increases β-cell proliferation and/or reduces β-cell apoptosis, which results in the preservation of β-cell mass [39,42,43]. Gene expression profiling studies have consistently shown that the expression of β-cell-associated transcription factors, such as *Mafa, Pdx1*, and *Nkx6.1*, is significantly higher in the islets of *db/db* mice treated with luseogliflozin, empagliflozin, and ipragliflozin than in those of untreated *db/db* mice [42,43,48,49,50]. Given that the expression of these transcription factors is lower in *db/db* mice than in wild-type mice and that they are involved in β-cell proliferation and apoptosis [27,51,52,53,54,55], the restoration of their expression by SGLT2i treatment may play an important role in the maintenance of β-cell mass induced by these drugs. Regarding dedifferentiation, in our study of *db/db* mice, there were no differences in the expression of markers of islet progenitor cells, including neurogenin 3 and aldehyde dehydrogenase 1a3, between luseogliflozin-treated and untreated mice [43]. Consistent with this, the SGLTi phloridzin does not reduce β-cell dedifferentiation in *db/db* mice [56]. However, one study has recently reported that treatment with dapagliflozin promotes α- to β-cell conversion and duct-derived β-cell neogenesis [57]. Thus, more detailed studies are needed to better define the effects of SGLT2is on the differentiation status of β-cells.

The effects of SGLT2is on metabolic stress, including oxidative stress, are considered to play a major role in the upstream mechanism, whereby the expression of the above-mentioned β-cell-associated transcription factors is restored [27,51,58,59]. SGLT2is likely reduce metabolic stress in β-cells, at least in part by reducing glucose overload (Figure 2), which leads to the restoration of the expression of these transcription factors. In fact, treatment with luseogliflozin and empagliflozin has been shown to increase the expression of the antioxidant gene *Gpx1* and to reduce that of *c-Jun*, which is located upstream of *Mafa* and induced by oxidative stress, in the islets of *db/db* mice [43,48,49]. Furthermore, in rodents with streptozotocin-induced type 1 diabetes, treatment with empagliflozin reduces ROS production and apoptosis in their β-cells [60]. Taken together, these findings are consistent with the above mechanism.

From a clinical point of view, when should the administration of SGLT2is be started during the course of T2D? To attempt to answer this question with respect to β-cell status, luseogliflozin was administered to *db/db* mice from various ages (6, 10, 14, or 24 weeks) for 4 weeks, and the β-cell mass of the mice was compared with those of mice that had not been administered luseogliflozin. The untreated *db/db* mice showed a decrease in β-cell mass with age, but treatment with luseogliflozin for 4 weeks increased the β-cell mass of the mice at all the assessed ages. However, treatment at a younger age preserved more of the initial β-cell mass [38]. Similarly, the effects of luseogliflozin treatment for 2 weeks on the β-cell mass and function of *db/db* mice during the early and advanced stages of diabetes were assessed. In this study, the insulin secretory capacity and β-cell mass of mice were preserved better when they were treated early (at 7–9 weeks of age) than when they were treated at an advanced stage (at 16–18 weeks) [49]. The results of these two studies imply that the protective effects of SGLT2is on β-cell mass and function are more marked during the earlier stages of diabetes. Another research group also investigated the importance of the duration and timing of treatment with dapagliflozin for the preservation of β-cell mass in *db/db* mice, and found that early long-term treatment is associated with superior protective effects [61]. Interestingly, when the mice were grouped into two groups with the same treatment period but with different starting times for the treatment with dapagliflozin, a β-cell protective effect was observed in mice in the early treatment group compared with mice in the late treatment group.

Because SGLT2is have similar molecular structures [62], the effect of these inhibitors on pancreatic β-cells is considered to be a class effect. However, it may be necessary to consider the effect of these inhibitors on pancreatic α-cells. SGLT2 has been reported to be expressed in α-cells and its inhibition secretes glucagon from α-cells [33] while other reports have shown that SGLT2 is not expressed in α-cells [34,35,36]. Therefore, the expression and role of SGLT2 in α-cells is highly controversial [63]. A recent report suggested that heterogeneity in SGLT2 expression and function in human α-cells was associated with interindividual variability [37]. Additionally, SGLT1 is expressed in α-cells and could contribute to the regulation of glucagon secretion from α-cells [35,64]. The finding that SGLT1 knockout mice fed a glucose-deficient fat-enriched diet showed altered islet morphology, including decreased insulin-expressing islet cells [65], suggested that the SGLT2 selectivity of SGLT2is affects β-cell mass and function via SGLT1 in α-cells (Figure 2). Future studies on a direct comparison of these inhibitors with different SGLT2 selectivity are required.

## 4. Clinical Findings Regarding the Effects of SGLT2is on Pancreatic β-Cell Function

Several clinical studies have investigated the effects of SGLT2is on β-cell function, although it is difficult to assess the direct effects on β-cell mass in individuals. Ferrannini et al. evaluated the responses to acute or chronic treatment with the SGLT2i empagliflozin in 66 individuals with T2D in a mechanistic single-arm study. In this study, β-cell glucose sensitivity, which was estimated using the data from a meal tolerance test, was compared as a β-cell function modeling. Both acute (single dose) and chronic (4-week administration) treatment with empagliflozin significantly increased the individuals’ β-cell glucose sensitivity versus baseline (prior to treatment) [66]. In addition, a randomized, double-blind, placebo-controlled phase 3 study of treatment with canagliflozin monotherapy and add-on therapy for 26 weeks showed improvements in β-cell glucose sensitivity and the insulin secretion rate versus placebo [67]. In a study of 24 individuals with T2D who were administered dapagliflozin or placebo for 2 weeks and underwent an oral glucose tolerance test and an euglycemic insulin clamp before and after treatment, not only β-cell glucose sensitivity but also the insulin secretion/insulin resistance index were significantly improved in the dapagliflozin group compared with the placebo group [68]. Furthermore, the β-cell function was investigated using the gold-standard hyperglycemic clamp technique in a single-arm study of 15 individuals with T2D who were administered empagliflozin for 2 weeks. This study also showed significant improvements in β-cell glucose sensitivity and the insulin secretion/insulin resistance index [69]. Finally, in a single-arm study of the effects of a 4-week treatment with ipragliflozin followed by a 1-week washout, in Japanese individuals with T2D, the insulin secretion/insulin resistance index significantly increased not only after the treatment for 4 weeks but also after a subsequent washout for 1 week compared with that before the treatment [70]. Taken together, these findings are consistent with a beneficial effect of SGLT2is on the β-cell function of individuals with T2D.

A high circulating concentration of proinsulin has been used as a marker of β-cell dysfunction [71]. The fasting proinsulin concentration is related to the level of glucose tolerance and can be measured in a single fasting blood sample, making it a convenient and clinically useful index [72,73]. In single-arm studies, the administration of ipragliflozin was shown to significantly reduce the proinsulin/C-peptide ratio in individuals with T2D [74,75]. This ratio was improved by canagliflozin monotherapy in similar individuals in two randomized, double-blind, placebo-controlled studies [76,77]. Given that a high circulating proinsulin concentration is indicative of a high β-cell workload [78], SGLT2is may prevent β-cell dysfunction by reducing the load on β-cells.

In addition to investigating the effects of empagliflozin on the β-cell function of individuals with diabetes, they have been investigated in individuals with impaired fasting glucose. Similar to the effects described above, treatment with empagliflozin for 2 days or 2 weeks significantly improved β-cell function, evaluated using the insulin secretion/insulin resistance index, in individuals with impaired fasting glucose [79]. In addition, a recent pooled analysis of data from the DAPA-HF and DAPA-CKD trials showed that treatment with dapagliflozin prevents the onset of diabetes in individuals with chronic kidney disease and HF [80]. Given that many of the individuals included in this analysis had prediabetes, the effect of SGLT2is to improve β-cell function may have contributed to the prevention of diabetes.

## 5. Comparison and Combination of SGLT2is and Other Antihyperglycemic Agents

Comparisons of the effects of SGLT2is and other antihyperglycemic agents and analyses of their concomitant effects on β-cell function have been made in several preclinical and clinical studies. To determine whether a reduction in metabolic demand induced by the antihyperglycemic effects or the insulin-sensitizing effects of the drugs are responsible for the improvements in β-cell function, 8-week-old *db/db* mice were treated for 4 weeks with liraglutide, a glucagon-like peptide 1 receptor (GLP-1R) agonist, alone or in combination with dapagliflozin, or rosiglitazone, a thiazolidinedione, alone or in combination with dapagliflozin. The results indicated that both combinations had synergistic effects to improve β-cell function, as evaluated in perifused isolated islets and using β-cell gene expression analysis [81].

The expression of GLP-1R in islets is downregulated in hyperglycemia and its expression is restored by correction of the hyperglycemia by treatment with phlorizin and luseogliflozin [49,82], and therefore it is reasonable to expect that therapy with a combination of an SGLT2i and an incretin-related agent would have a positive effect on β-cell function. In fact, combination treatment with empagliflozin and linagliptin for 2 weeks was significantly more effective at preserving β-cell mass and function than either drug when administered as a monotherapy in 7-week-old *db/db* mice [83]. In a clinical mechanistic study of individuals with T2D, glucose-stimulated insulin secretion, evaluated using an intravenous glucose load and a subsequent hyperglycemic clamp, was increased by treatment with empagliflozin, and a further increase was obtained after the addition of linagliptin [84]. In addition, a 24-week randomized placebo-controlled study showed that the proinsulin/C-peptide ratio in individuals with T2D was significantly reduced by the administration of canagliflozin as an add-on therapy to teneligliptin [85]. Finally, Ali et al. investigated the effect of treatment with canagliflozin, liraglutide, or a combination of these 2 agents on the β-cell function of individuals with T2D, and found that 16 weeks of monotherapy with canagliflozin or liraglutide improved β-cell glucose sensitivity, assessed by an oral glucose tolerance test. However, the combination therapy did not have an additional effect [86], which may be related to the lack of an additive antihyperglycemic effect of this combination.

Whether β-cell mass and function could be preserved by other agents with similarly sustained antihyperglycemic effects remained unclear. Therefore, 6-week-old *db/db* mice were treated with dapagliflozin alone, insulin glargine alone, or both for 8 weeks. In this study, the glucose tolerance was improved to the same extent in the three treatment groups compared with the control group. However, β-cell mass and function were preserved in the dapagliflozin and combination groups but not in the insulin glargine group [87]. The reason why β-cell mass and function were not preserved in mice treated with insulin glargine alone cannot be explained, despite the fact that all three treatment groups showed similar reductions in blood glucose concentrations. Although there were no differences in the lipid profiles of the three treatment groups, marked hepatic fat accumulation was present only in the mice treated with insulin glargine alone [87]. Given that hepatic steatosis is associated with β-cell dysfunction [88,89,90], this hepatic fat accumulation may have contributed to the difference in the effects of the treatments on β-cell mass and function. Further studies are needed to confirm this.

## 6. Future Prospects

As described above, many basic and clinical studies have demonstrated that SGLT2is preserve β-cell mass and function. In the future, the mechanism of the β-cell protective effect of SGLT2is should be studied in more detail. In particular, it is important to verify the effect of SGLT2i treatment on glucose metabolism and mitochondrial function in β-cells, to determine whether or not this treatment is consistent with the concept of a reduction in glucose metabolism overload, as discussed above. In addition, it is important to determine whether the β-cell protective effect of SGLT2is is solely because of a lowering of blood glucose. Recently, one study has reported that serum from mice treated with luseogliflozin increases β-cell proliferation not only in mouse islets but also in human islets [91]. These findings suggest that humoral factors other than blood glucose concentration may mediate the response of pancreatic β-cell mass to treatment with SGLT2is.

Clinically, it should be determined whether β-cell function can be maintained by long-term treatment with an SGLT2i and whether the effects of treatment can be maintained even after the discontinuation of the drug. It would also be interesting to investigate the effect of these drugs on the β-cell mass of humans using imaging techniques and also in individuals with prediabetes.
